# Quantity, topics, methods and findings of randomised controlled trials published by German university departments of general practice – systematic review

**DOI:** 10.1186/s13063-016-1328-y

**Published:** 2016-04-23

**Authors:** Stefan Heinmüller, Antonius Schneider, Klaus Linde, for the DFG Network Clinical Trials in General Practice

**Affiliations:** Institute of General Practice, University Hospital rechts der Isar,Technical University Munich, Orleansstrasse 47, 81667 Munich, Germany

**Keywords:** Germany, Primary care, General practice, Randomised controlled trials, Academic performance

## Abstract

**Background:**

Academic infrastructures and networks for clinical research in primary care receive little funding in Germany. We aimed to provide an overview of the quantity, topics, methods and findings of randomised controlled trials published by German university departments of general practice.

**Methods:**

We searched Scopus (last search done in April 2015), publication lists of institutes and references of included articles. We included randomised trials published between January 2000 and December 2014 with a first or last author affiliated with a German university department of general practice or family medicine. Risk of bias was assessed with the Cochrane tool, and study findings were quantified using standardised mean differences (SMDs).

**Results:**

Thirty-three trials met the inclusion criteria. Seventeen were cluster-randomised trials, with a majority investigating interventions aimed at improving processes compared with usual care. Sample sizes varied between 6 and 606 clusters and 168 and 7807 participants. The most frequent methodological problem was risk of selection bias due to recruitment of individuals after randomisation of clusters. Effects of interventions over usual care were mostly small (SMD <0.3). Sixteen trials randomising individual participants addressed a variety of treatment and educational interventions. Sample sizes varied between 20 and 1620 participants. The methodological quality of the trials was highly variable. Again, effects of experimental interventions over controls were mostly small.

**Conclusions:**

Despite limited funding, German university institutes of general practice or family medicine are increasingly performing randomised trials. Cluster-randomised trials on practice improvement are a focus, but problems with allocation concealment are frequent.

**Electronic supplementary material:**

The online version of this article (doi:10.1186/s13063-016-1328-y) contains supplementary material, which is available to authorized users.

## Background

Practice-based randomised controlled trials (RCTs) in primary care are essential, as they provide the basis for evidence-based decision-making in a central sector of health care [1]. Furthermore, being considered the gold standard for clinical research, high-quality RCTs led by general practitioners (GPs) are of crucial importance to enhance the still limited acceptance of general practice/family medicine as an academic discipline at German universities [2]. In recent years, several countries, such as the United Kingdom, the United States and the Netherlands, have invested greatly in the establishment of an efficient primary care research infrastructure (university departments of general practice or family medicine and practice networks) and the in practice-based RCTs [3–5].

Although Germany is Europe’s most populous country, its output of primary care research medicine lags far behind that of the United Kingdom and the Netherlands [3]. In 2000 only 5 of 36 German medical schools had a chair of general practice or family medicine, and by 2006 family medicine institutes or divisions had been established at 13 German universities [6]. By summer 2015, chairs had been established at 25 of 37 medical schools. Recently, a group of researchers from German academic departments of general practice published an analysis of obstacles in clinical trials [5]. In Germany, laboratory research is regarded more highly than clinical research. The single national funding programme for clinical research is highly competitive and specialist-dominated, and it usually favours innovation rather than comparative effectiveness research. General practice as an academic discipline is still not fully implemented, and most of the existing institutes are small. German GPs work in a market-oriented competitive system, mostly in small practices with a high caseload. Sustained funding for research-oriented practice networks is almost inexistent [5]. Despite these difficult circumstances, researchers in German university departments of general practice or family medicine have performed a number of randomised trials in recent years. Our aim in this article is to provide a descriptive overview of the current status of research productivity by performing a systematic review of the amount, topics, methods, quality and findings of randomised trials carried out by German university departments of general practice.

## Methods

The aims and basic methods we used to search the literature, establish the selection criteria and process, extract data, and assess risk of bias were predefined in an unpublished protocol (in German).

### Literature search

Publications were identified (1) by searching the Scopus database (http://info.scopus.com/; last searched 22 Apr 2015); (2) by screening publication lists of existing departments, institutes and divisions of general practice or family medicine at German medical schools; and (3) by tracking potentially relevant references in already-included articles. We selected Scopus as a database for electronic searching as it comprises PubMed/MEDLINE and also covers European journals in languages other than English, which are rarely listed in PubMed/MEDLINE. The following algorithm was used for our Scopus database search: AFFILCOUNTRY (deutschland) OR AFFILCOUNTRY (germany) AND AFFILORG (allgemeinmedizin) OR AFFILORG (general practice) AND PUBYEAR > 2009 AND PUBYEAR < 2015 AND (Random* OR Cluster). Publication lists were obtained directly from the departments, institutes and division and/or from their websites. Articles published until 2010 had been originally searched and identified for a previous review on any original research publication done by researchers at German academic family medicine departments [7, 8]. Articles published between 2011 and 2014 were identified by updated searches. (The year 2010 was also searched to detect trials potentially added to Scopus after completion of the search for our previous work.)

### Study selection

We included randomised (individual- or cluster-level) controlled trials published between January 2000 and December 2014 in which the first or last author of at least one relevant publication (study protocol and/or a publication reporting trial results) was affiliated with a general practice or family medicine department, institute or division of a German medical school. (For simplicity, only the term *department* is used in the rest of this article.) Within the overall project, we also collected published study protocols of RCTs for which results were not yet available by the end of 2014, but these are not included in the systematic review presented here. There were no predefined further exclusion criteria.

One reviewer screened titles and (to the extent available) abstracts of all Scopus search hits and excluded all clearly irrelevant publications. The full text was obtained for all remaining articles. For our previous review project [7], these were any articles potentially reporting original data. All articles actually reporting original data were then analysed for study topic and bibliographical and methodological characteristics. For our present analyses, only articles reporting on a prospective clinical trial with a control group or a protocol of such a study were considered as potentially relevant and checked as full texts. Titles and abstracts identified by our updated searches (2010–2014) were screened for randomised trials; publications clearly not reporting or related to a randomised trial were excluded. The first author checked all full texts obtained formally for compliance with our selection criteria. In cases of uncertainties, the senior author also read and assessed the articles.

### Data extraction

One reviewer extracted the following information (apart from reference information included in the Endnote file) from all included studies: study question in participants, intervention, control, outcome format; in case of a disease focus, the condition was recorded according to coding in the International Statistical Classification of Diseases and Related Health Problems, Tenth Revision, and the International Classification of Primary Care, Second Edition (http://www.who.int/classifications/icd/adaptations/icpc2/en/); information on authors; type and number of participating practices; the number of patients included, analysed and completing the studies, as well as information on recruitment; funding; study design issues, including duration, randomisation, blinding, sample size calculation and analysis; relevant outcome measures; and definition of a primary outcome measure.

### Assessment of risk of bias

Risk of bias was assessed using the risk of bias assessment tool of the Cochrane Collaboration [9] with items on random sequence generation, allocation concealment, blinding of participants and personnel, blinding of outcome assessment, incomplete outcome data, and selective reporting. Assessment was performed on the basis of the instructions given in the Cochrane Handbook [9] with a ‘rule book’ further standardising procedures, taking into account the great clinical and methodological diversity of the trials included in our review. Assessments were done by the first author. About half of the assessments were checked again by the senior author.

### Summarising the results of included trials

To provide a crude overview of the results of the included trials, we used both vote count methods and effect size calculations. For the vote count, the first author categorised overall study findings as ‘positive’ (findings in the intervention group consistently and statistically significantly better than in the control group), ‘trend positive’ (significant differences in favour of the intervention group only for some outcomes), ‘no difference’, ‘trend negative’ and ‘negative’ (as ‘trend positive’ and ‘positive’ but favouring the control group). Two vote counts were done: one based on what study authors reported and concluded and one according to the reviewer’s perception.

In addition, we calculated effect size estimates for predefined main outcome measures or, if a main outcome measure was not clearly defined, for the outcome we considered most relevant. Raw data in four formats (means, standard deviations and sample sizes; mean differences, samples sizes and *p* values or confidence intervals; events and number of participants per group; odds ratios and confidence intervals) were entered into a Comprehensive Meta-Analysis 3.3 spreadsheet (http://www.meta-analysis.com/index.php). This software allows conversion of different types of raw data into standardised mean differences (with 95 % confidence intervals). Positive values indicate better outcomes in the intervention group. We considered standardised mean differences ≤0.4 as small effects, between 0.41 and 0.7 as moderate effects and >0.7 as large effects [10].

## Results

**Fig. 1:**
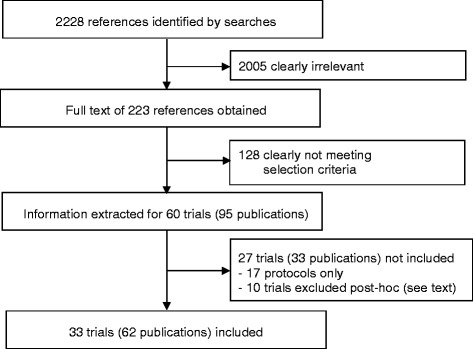
Flowchart of the study process

In our literature searches, we identified a total of 2228 references published between January 2000 and December 2014 (Fig. 1). On the basis of our review of titles and abstracts or the full-text check of articles which had already been identified for our previous review [7], a total of 2005 references were excluded as clearly irrelevant. Altogether, full texts of 223 publications were obtained. Of these, 128 were excluded because neither the first nor the last author was associated with a GP department, the studies were not randomised trials, or the authors reported additional information related to a randomised trial included which was not directly related to the main results (e.g., accompanying qualitative studies or cross-sectional analyses of baseline data). We extracted basic information on 60 studies described in 95 publications. Twenty-seven studies in 33 publication were excluded from the review (see Additional file 1 for references and Additional file 2 for study characteristics). For 17 trials, protocols (17 publications) were published between 2008 and 2014, but results were not available at the end of 2014. We decided post hoc to exclude a further 10 trials (16 publications) from the analyses for the present article because we considered them irrelevant to the primary aim of our overview. These studies comprised four trials, all comparing acupuncture with a sham acupuncture control having a first author with an affiliation with an institute of family medicine or general practice at the time of the publication for which we had definite information that the studies had actually been planned and performed when the author had worked at other departments; five which were short-term experimental trials focussed on physiological measures (e.g., effects of suggestion on pupil size) without any direct relation to the practice of family medicine; and one trial with a last author with an affiliation with a German institute of general practice but in which the study was performed exclusively in the United States.

Thirty-three trials (sixty-two publications) were included in the final analysis (Tables 1 and 2; see Additional file 1 for references). The number of trials increased sharply over time from 2 trials published between 2000 and 2004 to 10 trials published between 2005 and 2009 and 21 trials published between 2010 and 2014. Fifteen trials were funded by federal or regional ministries of education or health, eight trials received funding from a variety of other non-industry resources (e.g., foundations, social health insurance), two were industry-sponsored, and for eight the source of funding was not reported. We categorised 17 studies as cluster-randomised trials (trials in which the unit of randomisation and the level on which the outcome was measured differed; e.g., randomisation of practices and outcome measurement on the level of individual patients) and 16 trials as ‘normal’ randomised trials (randomisation of individuals with outcome measurement in the same individuals).

**Table 1:** Characteristics of included studies - cluster-randomised trials (*n* = 17)

First author, year	Participants’ conditions	Interventions	Controls	Main outcomes	Unit random.	Sample size^a^
Altiner, 2007	Cough	Educational intervention (GPs and patients)	No intervention	Antibiotic prescribing	Physician	104/2787
Becker, 2010	Low back pain	Group 1: Multifaceted guideline intervention	Postal dissemination of the guideline	Functional capacity	Practice	118/1378
		Group 2: 1 + training of practice nurses				
Erler, 2012	Elderly with chronic kidney disease	Multifaceted intervention helping adequate drug dosing	Usual care	Prescription exceeding recommended doses >30 %	Practice	46/404
Freiberger, 2013	Community-dwelling elderly	Risk of falls prevention program	No intervention	Risk of falling (main outcome falls not yet available)	Practice	33/378
Gensichen, 2009	Depression	Case management (communication, monitoring, behavioural activation)	Usual care	Depressive symptoms	Practice	74/626
Junius-Walker, 2012	Elderly GP patients	Training on structured priority-setting consultation	No training	Doctor-patient agreement on priorities	Practice	42/347
Kaufmann-Kolle, 2011	Asthma bronchiale	Quality circles with open benchmark	Traditional anonymous feedback in quality circles	Inappropriate drug combinations, asthma severity	Quality circle	6/unclear
Krones, 2008	All undergoing cholesterol measurement	Communication/shared decision-making in cardiovascular risk patients	Training on other subjects	Patient satisfaction, risk scores, participation	CME groups	14/1132
Mehring, 2013	Individuals with BMI ≥25 kg/m^2^	Web-based weight reduction program	Usual care	Weight reduction at 12 weeks	Practice	92/186
Mehring, 2014	Individuals willing to stop smoking	Web-based weight reduction program	Usual care	Biochemically confirmed smoking status at 12 weeks	Practice	92/168
Peters-Klimm, 2009	Chronic heart failure	Multifaceted, interdisciplinary medical educational intervention	Single 3-h lecture	Quality of life	Practice	37/168
Rosemann, 2007	Osteoarthritis	Group 1: Case management training GPs	No intervention	Quality of life (Arthritis Impact Measurement Scales Short Form)	Physician	75/1125
		Group 2: 1 + courses also for nurses				
Szecsenyi, 2012	Type 2 diabetes	Ideally implemented disease management	Usual disease management care	Achievement of target values for HbA1c and blood pressure	Practice	177/7807
Tinsel, 2013	Hypertension	Shared decision-making training	No training (usual care)	Patients’ perceived participation, blood pressure	Practice	37/1120
Vollmar, 2007	Dementia	Training in evidence-based dementia treatment (two slightly different groups)	Basic information (usual care)	Time to nursing home placement, death	Practice	303/390
Vollmar, 2010	GPs	Blended learning intervention on dementia care	Lecture and case discussion	Knowledge gain	Quality circle	26/305
Vormfelde, 2014	Patients receiving oral anticoagulation therapy	Educational program for patients provided by practice nurses	Brochure only	Knowledge, feelings about safety, complications	Practice	22/345

*Unit random.* unit of randomisation, *GP* general practitioner, *CME* continuing medical education, *HbA1c* haemoglobin A1c, BMI body mass index

See Additional file 1 for references

^a^For cluster-randomised trials, first number of clusters randomised/number of patients

**Table 2:** Characteristics of included studies: ‘normal’ randomised trials (*n* = 16)

First author, year	Participants/conditions	Interventions	Controls	Main outcomes	Unit random.	Sample size
Randomised trials investigating specific treatments (*n* = 11)						
Bleidorn, 2010	Uncomplicated urinary tract infection	Ibuprofen	Ciprofloxacin	Symptom resolution at day 4	Patient	80
Bücker, 2010	Acute, uncomplicated back pain	Handing out evidence-based back pain leaflet	Non-specific information	Functional capacity (Hannover Functional Ability Questionnaire)	Patient	189
du Moulin, 2009	COPD	Home-based exercise training	No intervention	6-minute walk test, quality of life, lung function	Patient	20
Frese, 2012	GP patients older than 70 years of age	Comprehensive geriatric assessment	Usual care	Mortality, nursing home admission	Patient	1620
Gastpar, 2003	Neurotic anxiety	Kava special extract WS 1490	Placebo	Anxiety Status Inventory	Patient	141
Hensler, 2009	Common cold	Intramuscular autologous blood therapy	Placebo	Duration of cold	Patient	139
Jobst, 2005	Recurrent respiratory infections	Intramuscular autologous blood injection	Homeopathic complex remedy (Engystol®; Biologische Heilmittel Heel, Baden-Baden, Germany)	Sick days	Patient	80
Klein, 2013	Non-specific neck pain	Strain-counterstrain (osteopathic technique)	Sham intervention	Range of motion and pain intensity	Patient	61
Peters-Klimm, 2010	Chronic heart failure	Case management (telephone monitoring and home visits)	Usual care	Quality of life, Kansas City Cardiomyopathy Questionnaire	Patient	199
Schencking, 2013	Osteoarthritis (hip or knee)	Group 1: Kneipp hydrotherapy	Both	Pain intensity, quality of life, mobility	Patient	30
		Group 2: Conventional physiotherapy				
Voigt, 2011	Migraine	Osteopathic manipulative treatment	No intervention	Migraine Disability Assessment (MIDAS), quality of life, pain	Patient	42
Randomised trials on other topics (*n* = 5)						
Bergold, 2013	First-year residents	Online course in evidence-based medicine	Wait list	Knowledge gain	First-year resident	120
Blank, 2013	Medical students	Additional near-peer teaching for physical examination	Established curricular course only	Objective structured clinical examination	Medical student	84
Butzlaff, 2004	General practitioners	Access to computerized guidelines	No specific access	Knowledge gain	Physician	72
Hoffmann, 2014	Physicians and nurses of GP practices	Team-based patient safety culture assessment and intervention	Short, facultative seminar on error management	Error management	Practice	65
Müller-Bühl, 2011	Adult GP patients	Answering three questions before completing the SF-36 quality of life questionnaire	Completing the SF-36 as usual	Number of missing items	Patient	215

*COPD* chronic obstructive pulmonary disease, *GP* general practitioner, *SF-36* 36-item Short Form Health Survey, *Unit random.* unit of randomisation

See Additional file 1 for references

### Cluster-randomised trials

The 17 cluster-randomised trials had a total of 37 study arms (14 two-armed and 3 three-armed trials; Table 2). Units of randomisation were quality circles or continuing medical education groups of several physicians in 3 studies, practices in 12 studies and individual physicians in 2 studies. Outcomes were measured on the level of individual patients in all but one study, in which the outcome was measured on the level of physicians. The number of randomised clusters varied between 6 and 303, and the number of included patients (physicians in 1 study) ranged between 168 and 7807. The conditions or clinical problems and outcomes assessed varied widely; no specific subject was investigated in more than one study. The majority of interventions were focused on the improvement of processes (managed care, more efficient or evidence-based strategies, better communication), and only a few were focused on defined, specific treatment strategies (e.g., a weight reduction program or a fall prevention intervention for the elderly). Control interventions were no intervention/usual care or minimal interventions unlikely to have relevant effects.

Many cluster-randomised trials were logistically complex and associated with high risk of bias (Table 3). While the generation of the random sequence was either adequate (ten studies) or not reported (seven studies), we considered the risk of selection bias related to allocation concealment high in ten studies, unclear in a further two and low in only five. The allocation of clusters was mostly concealed adequately. However, in those ten studies which received a high-risk rating, patients were explicitly or probably recruited *after* the practices knew their allocation status. Most authors seem to have been aware of this problem but were unable to manage recruitment otherwise. While in some trials this did not result in obvious imbalances, in others the number of patients recruited in the intervention and control groups differed beyond chance or there were clinically relevant differences in baseline characteristics of patients. Given the nature of the interventions tested, blinding of practices and individuals was not possible in any trial. While outcomes measured were partly objective, performance bias on the level of co-interventions cannot be ruled out. In five trials, a relevant proportion of randomised clusters did not recruit any patients and/or had a high percentage of incomplete outcome data, which resulted in a high risk of attrition bias. We considered the risk of major bias on the level of reporting outcomes to be low based on our (liberal) operationalization of this item. In summary, each of the cluster-randomised trials had a high risk of bias for at least one item.

**Table 3:** Risk of bias

First author, year	Sequence generation	Allocation concealment	Blinding of participants	Blinding of outcome assessment	Incomplete outcome data	Selective reporting
Cluster-randomised trials						
Altiner, 2007	Low	High	High	Low	High	Low
Becker, 2010	Low	High	High	High	High	Low
Erler, 2012	Low	Low	High	Low	Low	Low
Freiberger, 2013	Low	High	High	High	Unclear	Low
Gensichen, 2009	Low	High	High	High	Low	Low
Junius-Walker, 2012	Unclear	High	High	High	Unclear	Low
Kaufmann-Kolle, 2011	Unclear	High	High	Unclear	High	Low
Krones, 2008	Unclear	High	High	High	Unclear	Low
Peters-Klimm, 2009	Low	Low	High	Low	Unclear	Low
Mehring, 2013	Low	High	High	Low	Unclear	Low
Mehring, 2014	Low	High	High	Low	Unclear	Low
Rosemann, 2007	Unclear	Low	High	High	Unclear	Low
Szecsenyi, 2012	Unclear	Unclear	High	Low	Low	Low
Tinsel, 2013	Low	Low	Unclear	Unclear	High	Low
Vollmar, 2007	Unclear	High	High	Low	Low	Low
Vollmar, 2010	Unclear	Low	High	Unclear	High	Low
Vormfelde, 2014	Low	Unclear	High	High	Unclear	Low
Randomised trials investigating specific treatments						
Bleidorn, 2010	Low	Low	Low	Low	Low	Low
Bücker, 2010	Low	Low	High	High	High	Low
du Moulin, 2009	Low	Low	High	Low	Unclear	Low
Frese, 2012	Unclear	Unclear	Low	Low	High	Low
Gastpar, 2003	Low	Low	Low	Low	Low	Unclear
Hensler, 2009	Unclear	Low	Low	Low	Unclear	Low
Jobst, 2005	Low	Low	High	High	Unclear	Low
Klein, 2013	Low	Low	High	Low	Low	Low
Peters-Klimm, 2010	Low	Low	High	High	Unclear	High
Schencking, 2013	Low	Low	High	High	Low	Unclear
Voigt, 2011	Unclear	Unclear	High	High	Low	High
Randomised trials on other topics						
Bergold, 2013	Low	Low	High	High	Low	Low
Blank, 2013	Low	Low	High	Low	High	Low
Butzlaff, 2004	Low	Unclear	High	High	Low	Low
Hoffmann, 2014	Unclear	Unclear	High	Low	Low	Low
Müller-Bühl, 2011	Unclear	Unclear	Unclear	Unclear	Low	Low

See Additional file 1 for references

In the vote count, the conclusions of authors were categorised as ‘positive’ for five trials, as ‘trend positive’ in three and as ‘no difference’ in nine trials. The vote count based on the reviewer’s conclusion yielded similar categorisations (four, three and ten trials, respectively). Effect sizes estimates could be calculated for 15 trials with 18 comparisons of an intervention with a control group (Table 4). Only two trials had moderately large group differences, with standardised mean differences of −0.60 and −0.63, respectively. In all other trials, differences were small, ranging from −0.30 to 0.17. Confidence intervals did not include zero in eight trials.

**Table 4:** Effect size estimates

First author, year	Outcome used for effect size estimation	SMD	LL	UL
Cluster-randomised trials				
Altiner, 2007	Frequency of antibiotic prescription	0.30	0.14	0.46
Becker, 2010	Functional capacity 6 (group 1 vs. controls)	0.15	0.01	0.29
Becker, 2010	Functional capacity 6 (group 2 vs. controls)	0.11	−0.03	0.25
Erler, 2012	Number of patients with inadequate prescriptions	0.23	−0.11	0.56
Gensichen, 2009	Depression scores	0.22	0.05	0.38
Krones, 2008	Patient participation and satisfaction score	0.23	0.10	0.35
Peters-Klimm, 2009	Quality of life physical function	−0.17	−0.50	0.16
Rosemann, 2007	Arthritis impact, lower body (group 1 vs. controls)	0.08	−0.09	0.25
Rosemann, 2007	Arthritis impact, lower body (group 2 vs. controls)	0.17	0.00	0.34
Szecsenyi, 2012	Number of patients reaching treatment targets	0.01	−0.04	0.07
Tinsel, 2013	Shared decision-making score	0.07	−0.06	0.20
Vollmar, 2007	Institutionalisation (group 1 vs. controls)	0.08	−0.24	0.41
Vollmar, 2007	Institutionalisation (group 2 vs. controls)	−0.07	−0.38	0.25
Vormfelde, 2014	Knowledge scores anticoagulant treatment	0.63	0.41	0.85
Freiberger, 2013	Mobility	0.27	0.05	0.49
Mehring, 2013	Weight reduction	0.60	0.27	0.92
Mehring, 2014	Smoking cessation	0.08	−0.61	0.77
Vollmar, 2010	Knowledge gain	0.02	−0.28	0.33
Randomised trials investigating specific treatments				
Bücker, 2010	Functional capacity	0.28	−0.10	0.66
Peters-Klimm, 2010	Quality of life physical functioning	−0.04	−0.38	0.31
Bleidorn, 2010^a^	No symptom resolution	0.15	−0.37	0.68
du Moulin, 2009	6-minute walk test	1.03	0.10	1.97
Frese, 2012	Death	0.14	0.05	0.22
Gastpar, 2003	Anxiety scores	0.15	−0.18	0.48
Hensler, 2009	Illness duration	0.05	−0.32	0.41
Jobst, 2005^a^	Illness days	0.02	−0.42	0.46
Klein, 2013	Mobility restriction, neck	0.24	−0.27	0.74
Schencking, 2013	Pain (group 1 vs. controls)	0.20	−0.68	1.08
Schencking, 2013	Pain (group 2 vs. controls)	0.10	−0.78	0.97
Randomised trials on other topics				
Bergold, 2013	Knowledge of EBM	0.76	0.38	1.14
Blank, 2013	Score for clinical examination quality	1.16	0.58	1.74
Butzlaff, 2004	Knowledge gain	0.11	−0.43	0.65
Hoffmann, 2014	Error management	−0.06	−0.57	0.45
Müller-Bühl, 2011	Number of missing items	0.25	−0.02	0.52

*SMD* standardised mean difference, *LL* lower limit of the 95 % confidence interval, *UL* upper limit of the 95 % confidence interval

^a^Studies comparing two active treatments (equivalence or non-inferiority trials)

Data are presented as standardised mean differences with 95 % confidence intervals. Negative values indicate better outcomes in the intervention group.

See Additional file 1 for references

### ‘Normal’ randomised trials

In a total of 16 trials with 33 study arms (one 3-armed trial), the unit of randomisation and the level of outcome measurement were the same (Table 2). In 11 trials, researchers investigated the effectiveness of specific clinical interventions; these interventions were allocated to individual patients. Conditions as well as experimental and control interventions investigated were highly variable. Six of the eleven trials investigated complementary or alternative treatments (e.g., herbal drugs, autologous blood therapy, osteopathy or hydrotherapy). Sample sizes varied between 20 and 1620 patients. In the five remaining trials, researchers investigated educational interventions (three trials), the impact of access to computerized guidelines (one trial) and a methodological issue relevant to quality of life measurement in heterogeneous GP patient populations (one trial). The unit of randomisation was variable. Sample sizes varied between 72 and 215 participants.

In general, ‘normal’ randomised trials were logistically and methodologically less challenging than cluster-randomised trials. While not all trials reported details on sequence generation and allocation concealment, the risk of bias was never considered high. Instead, the risk of performance and measurement bias was considered high in the majority of studies due to the lack of blinding and/or use of subjective outcomes. Risk of bias due to incomplete outcome data was highly variable due to differences in challenges (some short-term studies did not have any attrition and had no or little missing data) and reporting or handling of the problems experienced. In two trials, there was clear evidence of biased reporting, either by selecting outcomes or by reporting inadequate analyses (focus on changes and inference testing within groups).

The authors’ conclusions were ‘positive’ in four trials, ‘trend positive’ in six and ‘no difference’ in six trials (reviewer’s conclusion three trials ‘positive’, five trials ‘trend positive’ and eight trials ‘no difference’). Standardised mean differences could be calculated for 15 trials including 16 comparisons (Table 4, lower part). Two trials actually compared active treatments. Large differences were reported in two trials and moderately large differences in one trial. In all other trials, point estimates of standardised mean differences were <0.3.

## Discussion

Despite limited funding, German university institutes for general practice or family medicine increasingly perform randomised trials; yet, the total number of 33 trials relevant to primary care published in a period of 15 years seems modest. In cluster-randomised trials, we noticed an emphasis on interventions aimed at improving processes in practices, while trials on drugs were very rare. The methodological quality of the trials was variable, with frequent relevant problems related to allocation concealment in cluster-randomised trials. Effects of the tested interventions over usual care or minimal interventions were mostly small.

It is somewhat difficult to compare the RCT output of university departments for general practice in Germany with that of other countries, as there is very limited information on such output internationally. The only systematic analysis limited to ‘RCTs with a general practice setting’ we found in the literature was published by Kortekaas et al. in 2012 [2]. These authors searched MEDLINE using the text words and/or MeSH (‘medical subject headings’) terms ‘general practice’, ‘primary healthcare’ or ‘family medicine’ to identify relevant trials published between 1990 and 2010. The 1935 publications on RCTs included 549 originated from the United States, 511 from the United Kingdom, 201 from Scandinavia, 194 from the Netherlands and 480 from a variety of other countries. The number of trials originating from Germany was not reported in the published review, but the first author kindly provided us an unpublished list of the 52 German studies. Of the 38 publications included by Kortekaas et al. that were published between 2000 and 2010, 8 were also included by us (while we identified additional 9 trials for this period), 3 were relevant protocol publications without results (also identified by us but excluded from the analysis presented), 1 was an additional publication on a trial already included in both reviews, 1 turned out not to be a randomised trial and 1 publication was a duplicate. The remaining 24 publications were not included by us because in 22 not a single author was affiliated with a GP department (or at least reported a private general practice as contact) and in 2 only a middle author had a GP department affiliation. This comparison shows that, depending on the objective and the methods used, bibliometric analyses can produce quite different findings. Yet, it makes clear that, compared with countries leading in primary care research (UK, USA, Netherlands, Scandinavian countries), Germany’s output of RCTs in the area of academic family medicine is rather low, and that many ‘primary care trials’ in Germany are conducted *with* or *on* GPs rather than *by* GPs.

There is a remarkable focus by German GP institutes on process-oriented cluster-randomised trials. Also, among the 16 protocols of trials without published results by 2014 identified by our search, 12 described cluster-randomised trials (see Additional file 2). This focus seems well compatible with the priorities in the research agenda of the European General Practice Research Network that ask for RCTs ‘which take into account broad issues such as patient preferences, multimorbidity, quality of life and social and environmental circumstances’ ([11] p. 11). Also, when we screened systematic reviews with the term *primary care* in the title in the Cochrane library (http://www.cochranelibrary.com/), it became clear that primary care research is focused more on complex and process interventions than on single specific interventions such as a defined drug. The methodological problems with allocation concealment and attrition observed in completed German cluster-randomised trials fit very well with the problems described in analyses of such trials in general [12–15]. It seems to us that the protocols of the partly very large newly planned or ongoing trials try to take these problems and former experiences into account. We are not aware of any systematic analyses of effect sizes in general practice trials across conditions, but it seems plausible to us that, in the primary care setting with its multiple influence factors, intervention effects are often small. Yet, for some of the interventions tested in the trials reviewed by us, we wondered whether these were conceptually really promising and/or well implemented.

When interpreting our findings, it must be kept in mind that we searched and included only trials in which the first or last authors were affiliated with a German university GP department. Therefore, our results cannot be adopted for RCTs authored by GPs without such an affiliation. However, our check of the additional publications identified by Kortekaas et al. [2] suggests the number of such studies is small. We excluded ten trials post hoc as they either were conducted when authors were working at other departments (and only written up later) or had no thematic relation to primary care whatsoever. We think these exclusions were necessary to allow for a judgement of the real RCT output of German academic GP departments relevant for their field. Due to limited resources, many working steps in this review were performed by a single author, with only some checks done by a second author. Therefore, some extraction or assessment errors might have gone undetected. The standardised mean differences calculated by us should be interpreted only as crude indicators of how large differences between groups were and should not be used for clinical decision-making.

## Conclusions

Researchers in Germany’s academic departments of general practice and family medicine increasingly perform and publish RCTs. However, without increased and sustained funding for research infrastructure and single trial projects, there will be little chance to catch up with leading countries such as the United Kingdom, the Netherlands, Scandinavian countries or the United States. We hope that our analysis will help to avoid some preventable shortcomings in future trials.

## Additional files

Additional file 1: References of included and excluded trials. (DOCX 32 kb) Additional file 2: Details of excluded studies. (DOCX 17 kb)

## Abbreviations

BMI: body mass index
CME: continuing medical education
COPD: chronic obstructive pulmonary disease
EBM: evidence-based medicine
GP: general practitioner
HbA1c: haemoglobin A1c
LL: lower limit of the 95 % confidence interval
RCT: randomised controlled trial
SF-36: 36-item Short Form Health Survey
SMD: standardised mean difference
UL: upper limit of the 95 % confidence interval
