# Supplementary material for: Quantity, topics, methods and findings of randomised controlled trials published by German university departments of general practice – systematic review
Source: Trials. 2016 Apr 23;17:211. doi: 10.1186/s13063-016-1328-y (PMC4842270; doi:10.1186/s13063-016-1328-y)
Supplement: Additional file 1: — References of included and excluded trials. (DOCX 32 kb) [file 13063_2016_1328_MOESM1_ESM.docx]

**References of included and excluded trials**

**References of included trials**

In case of more than one publication for a trial: *main publication; **publication with GP first and/or last author other than the main publication; (P) protocol

**Cluster-randomized trials**

Altiner 2007

Altiner A, Brockmann S, Sielk M, Wilm S, Wegscheider K, Abholz HH: Reducing antibiotic prescriptions for acute cough by motivating GPs to change their attitudes to communication and empowering patients: A cluster-randomized intervention study. Journal of Antimicrobial Chemotherapy 2007, 60(3):638-644.

Becker 2010

*Becker A, Held H, Redaelli M, Strauch K, Chenot JF, Leonhardt C, et al: Low back pain in primary care: Costs of care and prediction of future health care utilization. Spine 2010, 35(18):1714-1720.

Becker A, Held H, Redaelli M, Chenot JF, Leonhardt C, Keller S, et al: Implementation of a guideline for low back pain management in primary care: A cost-effectiveness analysis. Spine 2012, 37(8):701-710.

Leonhardt C, Keller S, Chenot JF, Luckmann J, Basler HD, Wegscheider K, et al: TTM-based motivational counselling does not increase physical activity of low back pain patients in a primary care setting-A cluster-randomized controlled trial. Patient Education and Counseling 2008, 70(1):50-60.

Erler 2012

Erler A, Beyer M, Petersen JJ, Saal K, Rath T, Rochon J, et al: How to improve drug dosing for patients with renal impairment in primary care - A cluster-randomized controlled trial. BMC Family Practice 2012, 13:91.

Freiberger 2013

**Blank WA, Freiberger E, Siegrist M, Landendoerfer P, Linde K, Schuster T, et al: An interdisciplinary intervention to prevent falls in community-dwelling elderly persons: Protocol of a cluster-randomized trial [PreFalls]. BMC Geriatrics 2011, 11. (P)

*Freiberger E, Blank WA, Salb J, Geilhof B, Hentschke C, Landendoerfer P, et al: Effects of a complex intervention on fall risk in the general practitioner setting: A cluster randomized controlled trial. Clinical Interventions in Aging 2013, 8:1079-1088.

Gensichen 2009

*Gensichen J, Von Korff M, Peitz M, Muth C, Beyer M, Güthlin C, et al: Case management for depression by health care assistants in small primary care practices: A cluster randomized trial. Annals of Internal Medicine 2009, 151(6):369-378.

Gensichen J, Petersen JJ, Von Korff M, Heider D, Baron S, Kon̈ig J, et al: Cost-effectiveness of depression case management in small practices. British Journal of Psychiatry 2013, 202(6):441-446.

Gensichen J, Petersen JJ, Karroum T, Rauck S, Ludman E, König J, et al: Positive impact of a family practice-based depression case management on patient's self-management. General Hospital Psychiatry 2011, 33(1):23-28.

Gensichen J, Torge M, Peitz M, Wendt-Hermainski H, Beyer M, Rosemann T, et al: Case management for the treatment of patients with major depression in general practices - Rationale, design and conduct of a cluster randomized controlled trial - PRoMPT (Primary care Monitoring for depressive Patient's Trial) [ISRCTN66386086] - Study protocol. BMC Public Health 2005, 5:101. (P)

Petersen JJ, König J, Paulitsch MA, Mergenthal K, Rauck S, Pagitz M, et al: Long-term effects of a collaborative care intervention on process of care in family practices in Germany: A 24-month follow-up study of a cluster randomized controlled trial. General Hospital Psychiatry 2014, 36(6):570-574.

Junius-Walker 2012

*Junius-Walker U, Wrede J, Voigt I, Hofmann W, Wiese B, Hummers-Pradier E, et al: Impact of a priority-setting consultation on doctor-patient agreement after a geriatric assessment: Cluster randomised controlled trial in German general practices. Quality in Primary Care 2012, 20(5):321-334.

Wrede J, Voigt I, Bleidorn J, Hummers-Pradier E, Dierks ML, Junius-Walker U: Complex health care decisions with older patients in general practice: Patient-centeredness and prioritization in consultations following a geriatric assessment. Patient Education and Counseling 2013, 90(1):54-60.

Voigt I, Wrede J, Diederichs-Egidi H, Dierks ML, Hummers-Pradier E, Junius-Walker U: PräfCheck: Patientenzentrierte Behandlungsplanung mit älteren multimorbiden Patienten. Zeitschrift für Gerontologie und Geriatrie 2010, 43(5):303-309. (P)

Kaufmann-Kolle 2011

*Kaufmann-Kolle P, Szecsenyi J, Broge B, Haefeli WE, Schneider A: Führt die Implementierung von offenem Benchmarking in datengestützten Qualitätszirkeln zur Verbesserung der hausärztlichen Versorgung bei Arzneimittelinteraktion und Asthma bronchiale? ZEFQ 2011, 105(5):389-395.

Schneider A, Wensing M, Biessecker K, Quinzler R, Kaufmann-Kolle P, Szecsenyi J: Impact of quality circles for improvement of asthma care: Results of a randomized controlled trial. Journal of Evaluation in Clinical Practice 2008, 14(2):185-190.

Krones 2008

Hirsch O, Keller H, Albohn-Kühne C, Krones T, Donner-Banzhoff N: Satisfaction of patients and primary care physicians with shared decision making. Evaluation and the Health Professions 2010, 33(3):321-342.

Keller H, Hirsch O, Kaufmann-Kolle P, Krones T, Becker A, Sönnichsen AC, et al: Evaluating an implementation strategy in cardiovascular prevention to improve prescribing of statins in Germany: An intention to treat analysis. BMC Public Health 2013, 13:623.

Keller H, Krones T, Becker A, Hirsch O, Sönnichsen AC, Popert U, et al: Arriba: Effects of an educational intervention on prescribing behaviour in prevention of CVD in general practice. European Journal of Preventive Cardiology 2012, 19(3):322-329.

*Krones T, Keller H, Sönnichsen A, Sadowski EM, Baum E, Wegscheider K, et al: Absolute cardiovascular disease risk and shared decision making in primary care: A randomized controlled trial. Annals of Family Medicine 2008, 6(3):218-227.

Krones T, Keller H, Becker A, Sönnichsen A, Baum E, Donner-Banzhoff N: The theory of planned behaviour in a randomized trial of a decision aid on cardiovascular risk prevention. Patient Education and Counseling 2010, 78(2):169-176.

Mehring 2013

Mehring M, Haag M, Linde K, Wagenpfeil S, Frensch F, Blome J, et al: Effects of a general practice guided web-based weight reduction program - Results of a cluster-randomized controlled trial. BMC Family Practice 2013, 14:76.

Mehring 2014

Mehring M, Haag M, Linde K, Wagenpfeil S, Schneider A: Effects of a guided web-based smoking cessation program with telephone counseling: a cluster randomized controlled trial. J Med Internet Res 2014, 16(9):e218.

Peters-Klimm 2009

*Peters-Klimm F, Campbell S, Müller-Tasch T, Schellberg D, Gelbrich G, Herzog W, et al: Primary care-based multifaceted, interdisciplinary medical educational intervention for patients with systolic heart failure: Lessons learned from a cluster randomised controlled trial. Trials 2009, 10:68.

Peters-Klimm F, Müller-Tasch T, Remppis A, Szecsenyi J, Schellberg D: Improved guideline adherence to pharmacotherapy of chronic systolic heart failure in general practice - Results from a cluster-randomized controlled trial of implementation of a clinical practice guideline. Journal of Evaluation in Clinical Practice 2008, 14(5):823-829.

Peters-Klimm F, Laux G, Campbell S, Müller-Tasch T, Lossnitzer N, Schultz JH, et al: Physician and patient predictors of evidence-based prescribing in heart failure: A multilevel study. PLoS ONE 2012, 7(2):e31082.

Rosemann 2007

*Rosemann T, Joos S, Laux G, Gensichen J, Szecsenyi J: Case management of arthritis patients in primary care: A cluster-randomized controlled trial. Arthritis Care and Research 2007, 57(8):1390-1397.

Rosemann T, Körner T, Wensing M, Gensichen J, Muth C, Joos S, et al: Rationale, design and conduct of a comprehensive evaluation of a primary care based intervention to improve the quality of life of osteoarthritis patients. The PraxArt-project: A cluster randomized controlled trial [ISRCTN87252339]. BMC Public Health 2005, 5: 77. (P)

Szecsenyi 2012

*Evaluation des DMP Diabetes mellitus Typ 2 im Rahmen der ELSID-Studie, Abschlussbericht für den AOK-Bundesverband [http://www.aok-gesundheitspartner.de/imperia/md/gpp/bund/dmp/evaluation/elsid/dmp_elsid_abschlussbericht_2012.pdf]

Joos S, Rosemann T, Heiderhoff M, Wensing M, Ludt S, Gensichen J, et al: ELSID-Diabetes study-evaluation of a large scale implementation of disease management programmes for patients with type 2 diabetes. Rationale, design and conduct - A study protocol [ISRCTN08471887]. BMC Public Health 2005, 5:99. (P)

Tinsel 2013

*Tinsel I, Buchholz A, Vach W, Siegel A, Dürk T, Buchholz A, et al: Shared decision-making in antihypertensive therapy: A cluster randomised controlled trial. BMC Family Practice 2013, 14.

Tinsel I, Buchholz A, Vach W, Siegel A, Dürk T, Loh A, et al: Implementation of shared decision making by physician training to optimise hypertension treatment. Study protocol of a cluster-RCT. BMC Cardiovascular Disorders 2012, 12:72. (P)

Vollmar 2007

Donath C, Grassel E, Grossfeld-Schmitz M, Menn P, Lauterberg J, Wunder S, et al: Effects of general practitioner training and family support services on the care of home-dwelling dementia patients--results of a controlled cluster-randomized study. BMC Health Services Research 2010, 10:314.

Holle R, Gräßel E, Ruckdäschel S, Wunder S, Mehlig H, Marx P, et al: Dementia care initiative in primary practice study protocol of a cluster randomized trial on dementia management in a general practice setting. BMC Health Services Research 2009, 9:91. (P)

Lauterberg J, Großfeld-Schmitz M, Ruckdäschel S, Neubauer S, Mehlig H, Gaudig M, et al: Projekt IDA - Konzept und Umsetzung einer cluster-randomisierten Studie zur Demenzversorgung im hausärztlichen Bereich. ZaeFQ 2007, 101(1):21-26.

*Menn P, Holle R, Kunz S, Donath C, Lauterberg J, Leidl R, et al: Dementia care in the general practice setting: A cluster randomized trial on the effectiveness and cost impact of three management strategies. Value in Health 2012, 15(6):851-859.

**Vollmar HC, Gräßel E, Lauterberg J, Neubauer S, Großfeld-Schmitz M, Koneczny N, et al: Multimodale Schulung von Hausärzten - Evaluation und Wissenszuwachs im Rahmen der Initiative Demenzversorgung in der Allgemeinmedizin (IDA). ZEFQ 2007, 101(1):27-34.

Vollmar 2010

Vollmar HC, Butzlaff ME, Lefering R, Rieger MA: Knowledge translation on dementia: A cluster randomized trial to compare a blended learning approach with a "classical" advanced training in GP quality circles. BMC Health Services Research 2007, 7:92. (P)

*Vollmar HC, Mayer H, Ostermann T, Butzlaff ME, Sandars JE, Wilm S, et al: Knowledge transfer for the management of dementia: A cluster-randomised trial of blended learning in general practice. Implementation Science 2010, 5:1.

Vollmar HC, Mayer H, Rieger M, Wilm S, Ostermann T: Blended Learning zur hausärztlichen Demenz-Fortbildung: Einschätzung durch Nutzer einer cluster-randomisierten Studie. GMS Medizinische Informatik, Biometrie und Epidemiologie 2010, 6(2):1 - 8.

Vormfelde 2014

Hua TD, Vormfelde SV, Abed MA, Schneider-Rudt H, Sobotta P, Friede T, et al: Practice nursed-based, individual and video-assisted patient education in oral anticoagulation - Protocol of a cluster-randomized controlled trial. BMC Family Practice 2011, 12.:17. (P)

*Vormfelde SV, Abu Abed M, Hua TD, Schneider S, Friede T, Chenot JF: Educating orally anticoagulated patients in drug safety: A cluster-randomized study in general practice. Dtsch Arztebl Inter 2014, 111(37):607-614.

**Randomized trials investigating specific treatments**

Bleidorn 2010

*Bleidorn J, Gágyor I, Kochen MM, Wegscheider K, Hummers-Pradier E: Symptomatic treatment (ibuprofen) or antibiotics (ciprofloxacin) for uncomplicated urinary tract infection? - Results of a randomized controlled pilot trial. BMC Medicine 2010, 8:30.

Gágyor I, Bleidorn J, Wegscheider K, Hummers-Pradier E, Kochen MM: Practices, patients and (im)perfect data - feasibility of a randomised controlled clinical drug trial in German general practices. Trials 2011, 12.

Bücker 2010

Bücker B, Butzlaff M, Isfort J, Koneczny N, Vollmar HC, Lange S, et al: Patienteninformationen und Kreuzschmerzen (PIK-Studie) - Einfluss von Patienteninformationen auf Wissen und Funktionskapazität von Patienten mit akuten, unkomplizierten Rückenschmerzen. Gesundheitswesen 2010, 72(12):e78-e88.

Du Moulin 2010

Du Moulin M, Taube K, Wegscheider K, Behnke M, Van Den Bussche H: Home-based exercise training as maintenance after outpatient pulmonary rehabilitation. Respiration 2009, 77(2):139-145.

Frese 2012

Frese T, Deutsch T, Keyser M, Sandholzer H: In-home preventive comprehensive geriatric assessment (CGA) reduces mortality--a randomized controlled trial. Archives of gerontology and geriatrics 2012, 55(3):639-644.

Gastpar 2003

Gastpar M, Klimm HD: Treatment of anxiety, tension and restlessness states with Kava special extract WS® 1490 in general practice: A randomized placebo-controlled double-blind multicenter trial. Phytomedicine 2003, 10(8):631-639.

Hensler 2009

Hensler S, Guendling PW, Schmidt M, Jork K: Autologous blood therapy for common cold-A randomized, double-blind, placebo-controlled trial. Complementary Therapies in Medicine 2009, 17(5-6):257-261.

Jobst 2005

Jobst D, Altiner A, Wegscheider K, Abholz HH: Helfen intramuskuläre eigenblutgaben bei chronisch rezidivierenden infekten der atemwege? - Fußangeln auf dem weg einer randomisierten studie. ZFA 2005, 81(6):258-263.

Klein 2013

Klein R, Bareis A, Schneider A, Linde K: Strain-counterstrain to treat restrictions of the mobility of the cervical spine in patients with neck pain-A sham-controlled randomized trial. Complementary Therapies in Medicine 2013, 21(1):1-7.

Peters-Klimm 2010

Freund T, Baldauf A, Muth C, Gensichen J, Szecsenyi J, Peters-Klimm F: Praxisbasiertes Hausbesuchs- und Telefonmonitoring von Patienten mit Herzinsuffizienz: Rationale, Design und praktische Anwendung der Monitoringslisten in der HICMan-Studie. ZEFQ 2011, 105(6):434-445.

*Peters-Klimm F, Campbell S, Hermann K, Kunz CU, Müller-Tasch T, Szecsenyi J: Case management for patients with chronic systolic heart failure in primary care: The HICMan exploratory randomised controlled trial. Trials 2010, 11:56.

Peters-Klimm F, Müller-Tasch T, Schellberg D, Gensichen J, Muth C, Herzog W, et al: Rationale, design and conduct of a randomised controlled trial evaluating a primary care-based complex intervention to improve the quality of life of heart failure patients: HICMan (Heidelberg Integrated Case Management). BMC Cardiovascular Disorders 2007, 7:25. (P)

Schencking 2013

Schencking M, Otto A, Deutsch T, Sandholzer H: A comparison of Kneipp hydrotherapy with conventional physiotherapy in the treatment of osteoarthritis of the hip or knee: Protocol of a prospective randomised controlled clinical trial. BMC Musculoskeletal Disorders 2009, 10:104. (P)

*Schencking M, Wilm S, Redaelli M: A comparison of Kneipp hydrotherapy with conventional physiotherapy in the treatment of osteoarthritis: A pilot trial. Journal of integrative medicine 2013, 11(1):17-25.

Voigt 2011

Voigt K, Liebnitzky J, Burmeister U, Sihvonen-Riemenschneider H, Beck M, Voigt R, et al: Efficacy of osteopathic manipulative treatment of female patients with migraine: Results of a randomized controlled trial. Journal of Alternative and Complementary Medicine 2011, 17(3):225-230.

**Randomized trials on other topics**

Bergold 2013

Bergold M, Strametz R, Weinbrenner S, Khan KS, Zamora J, Moll P, Er at: Evidence-based Medicine online for young doctors-a randomised controlled trial. ZEFQ 2013, 107(1):36-43.

Blank 2013

Blank WA, Blankenfeld H, Vogelmann R, Linde K, Schneider A: Can near-peer medical students effectively teach a new curriculum in physical examination? BMC Medical Education 2013, 13:165.

Butzlaff 2014

Butzlaff M, Vollmar HC, Floer B, Koneczny N, Isfort J, Lange S: Learning with computerized guidelines in general practice?: A randomized controlled trial. Family Practice 2004, 21(2):183-188.

Hoffmann 2014

Hoffmann B, Müller V, Rochon J, Gondan M, Müller B, Albay Z, et al: Effects of a team-based assessment and intervention on patient safety culture in general practice: An open randomised controlled trial. BMJ Quality and Safety 2014, 23(1):35-46.

Müller Bühl 2011

Müller-Bühl U, Franke B, Hermann K, Engeser P: Lowering missing item values in quality-of-life questionnaires: An interventional study. International Journal of Public Health 2011, 56(1):63-69.

**References of excluded trials**

**Protocols without results published by Dec-31-2014**

Altiner 20012a

Altiner A, Berner R, Diener A, Feldmeier G, Köchling A, Löffler C, Schröder H, Siegel A, Wollny A, Kern WV: Converting habits of antibiotic prescribing for respiratory tract infections in German primary care - The cluster-randomized controlled CHANGE-2 trial. BMC Family Practice 2012, 13.

Altiner 2012b

Altiner A, Schafer I, Mellert C, Loffler C, Mortsiefer A, Ernst A, Stolzenbach CO, Wiese B, Scherer M, van den Bussche H et al: Activating GENeral practitioners dialogue with patients on their Agenda (MultiCare AGENDA) study protocol for a cluster randomized controlled trial. BMC Family Practice 2012, 13:118.

Barzel 2013

Barzel A, Ketels G, Tetzlaff B, Kruger H, Haevernick K, Daubmann A, Wegscheider K, Scherer M: Enhancing activities of daily living of chronic stroke patients in primary health care by modified constraint-induced movement therapy (HOMECIMT): study protocol for a cluster randomized controlled trial. Trials 2013, 14:334.

Bozorgmehr 2014

Bozorgmehr K, Szecsenyi J, Ose D, Besier W, Mayer M, Krisam J, Jacke CO, Salize HJ, Brandner R, Schmitt S et al: Practice network-based care management for patients with type 2 diabetes and multiple comorbidities (GEDIMAplus): Study protocol for a randomized controlled trial. Trials 2014, 15:243.

Drewelow 2012

Drewelow E, Wollny A, Pentzek M, Immecke J, Lambrecht S, Wilm S, Schluckebier I, Löscher S, Wegscheider K, Altiner A: Improvement of primary health care of patients with poorly regulated diabetes mellitus type 2 using shared decision-making - The DEBATE trial. BMC Family Practice 2012, 13:88.

Freund 2011

Freund T, Peters-Klimm F, Rochon J, Mahler C, Gensichen J, Erler A, Beyer M, Baldauf A, Gerlach FM, Szecsenyi J: Primary care practice-based care management for chronically ill patients (PraCMan): Study protocol for a cluster randomized controlled trial [ISRCTN56104508]. Trials 2011, 12:163.

Gágyor 2012

Gágyor I, Hummers-Pradier E, Kochen MM, Schmiemann G, Wegscheider K, Bleidorn J: Immediate versus conditional treatment of uncomplicated urinary tract infection - a randomized-controlled comparative effectiveness study in general practices. BMC infectious diseases 2012, 12:146.

Gensichen 2014

Gensichen J, Hiller TS, Breitbart J, Teismann T, Brettschneider C, Schumacher U, Piwtorak A, König HH, Hoyer H, Schneider N et al: Evaluation of a practice team-supported exposure training for patients with panic disorder with or without agoraphobia in primary care - study protocol of a cluster randomised controlled superiority trial. Trials 2014, 15:112.

Gummersbach 2013

Gummersbach E, In der Schmitten J, Abholz HH, Wegscheider K, Pentzek M: Effects of different information brochures on women's decision-making regarding mammography screening: Study protocol for a randomized controlled questionnaire study. Trials 2013, 14:319.

Jäger 2014

Jäger C, Freund T, Steinhäuser J, Joos S, Wensing M, Szecsenyi J: A tailored implementation intervention to implement recommendations addressing polypharmacy in multimorbid patients: Study protocol of a cluster randomized controlled trial. Trials 2013, 14:420.

Löffler 2014a

Löffler C, Böhmer F, Hornung A, Lang H, Burmeister U, Podbielski A, Wollny A, Kundt G, Altiner A: Dental care resistance prevention and antibiotic prescribing modification-the cluster-randomised controlled DREAM trial. Implementation Science 2014, 9:27.

Löffler 2014b

Löffler C, Drewelow E, Paschka SD, Frankenstein M, Eger J, Jatsch L, Reisinger EC, Hallauer JF, Drewelow B, Heidorn K et al: Optimizing polypharmacy among elderly hospital patients with chronic diseases-study protocol of the cluster randomized controlled POLITE-RCT trial. Implementation Science 2014, 9:151.

Mortsiefer 2008 (results published 2015)

Mortsiefer A, Meysen T, Schumacher M, Lintges C, Stamer M, Schmacke N, Wegscheider K, Abholz HH, In Der Schmitten J: CRISTOPH - A cluster-randomised intervention study to optimise the treatment of patients with hypertension in General Practice. BMC Family Practice 2008, 9:33.

Schmidt 2010

Schmidt CO, Pfingsten M, Fahland RA, Lindena G, Marnitz U, Pfeifer K, Kohlmann T, Chenot JF: Assessing a risk tailored intervention to prevent disabling low back pain - Protocol of a cluster randomized controlled trial. BMC Musculoskeletal Disorders 2010, 11:5.

Schmidt 2014

Schmidt K, Thiel P, Mueller F, Schmuecker K, Worrack S, Mehlhorn J, Engel C, Brenk-Franz K, Kausche S, Jakobi U et al: Sepsis survivors monitoring and coordination in outpatient health care (SMOOTH): Study protocol for a randomized controlled trial. Trials 2014, 15:283.

Siebenhofer 2012

Siebenhofer A, Ulrich LR, Mergenthal K, Roehl I, Rauck S, Berghold A, Harder S, Gerlach FM, Petersen JJ: Primary care management for optimized antithrombotic treatment [PICANT]: study protocol for a cluster-randomized controlled trial. Implementation Science 2012, 7:79.

Zimmermann 2014

Zimmermann T, Puschmann E, Ebersbach M, Daubmann A, Steinmann S, Scherer M: Effectiveness of a primary care based complex intervention to promote self-management in patients presenting psychiatric symptoms: study protocol of a cluster-randomized controlled trial. BMC Psychiatry 2014, 14:2.

**Trials excluded for various reasons**

Doering 2001

Doering TJ, Thiel J, Steuernagel B, Johannes B, Konitzer M, Niederstadt C, et al: Veränderung kognitiver Hirnleistungen im Alter durch Kneippanwendungen. Forschende Komplementärmedizin und Klassische Naturheilkunde 2001, 8(2):80-84.

Doering 2002

Doering TJ, Konitzer M, Hausner T, Steuernagel B, Schneider B, Fischer GC: Zerebrale Hämodynamik bei Kohlendioxidapplikationen. Forschende Komplementärmedizin und Klassische Naturheilkunde 2002, 9(3):148-152.

Gawrylyuk 2010

Gavrylyuk G, Ehrt O, Meissner K: Keine Effekte von placeboinduzierten Erwartungen auf die Pupillengröße und die Augenakkommodation. Zeitschrift fur Medizinische Psychologie 2010, 19(3-4):154-160.

Joos 2005

*Joos S, Brinkhaus B, Maluche C, Maupai N, Kohnen R, Kraehmer N, et al: Acupuncture and moxibustion in the treatment of active Crohn's disease: a randomized controlled study. Digestion 2004, 69(3):131-139.

**Joos S, Brinkhaus B, Maluche C, Maupai N, Kohnen R, Hahn E, et al: Akupunktur und Moxibustion als ergänzende Therapie bei M. Crohn. Deutsche Zeitschrift für Akupunktur 2005, 48(1):6-17.

Joos 2006

Joos S, Wildau N, Kohnen R, Szecsenyi J, Schuppan D, Willich SN, et al: Acupuncture and moxibustion in the treatment of ulcerative colitis: a randomized controlled study. Scandinavian Journal of Gastroenterology 2006, 41(9):1056-1063.

Ludman 2013

*Katon WJ, Lin EH, Von Korff M, Ciechanowski P, Ludman EJ, Young B, et al: Collaborative care for patients with depression and chronic illnesses. New England Journal of Medicine 2010, 363(27):2611-2620.

Lin EH, Von Korff M, Ciechanowski P, Peterson D, Ludman EJ, Rutter CM, et al: Treatment adjustment and medication adherence for complex patients with diabetes, heart disease, and depression: a randomized controlled trial. Annals of Family Medicine 2012, 10(1):6-14.

**Ludman EJ, Peterson D, Katon WJ, Lin EHB, Von Korff M, Ciechanowski P,et al: Improving confidence for self care in patients with depression and chronic illnesses. Behavioral Medicine 2013, 39(1):1-6.

Meissner 2011

Meissner K, Ziep D: Organ-specificity of placebo effects on blood pressure. Autonomic Neuroscience: Basic and Clinical 2011, 164(1-2):62-66.

Ronel 2011

Ronel J, Mehilli J, Ladwig KH, Blättler H, Oversohl N, Byrne RA, et al: Effects of verbal suggestion on coronary arteries: Results of a randomized controlled experimental investigation during coronary angiography. American Heart Journal 2011, 162(3):507-511.

Schneider 2005

**Schneider A, Löwe B, Streitberger K: Perception of bodily sensation as a predictor of treatment response to acupuncture for postoperative nausea and vomiting prophylaxis. Journal of Alternative and Complementary Medicine 2005, 11(1):119-125.

*Streitberger K, Diefenbacher M, Bauer A, Conradi R, Bardenheuer H, Martin E, et al: Acupuncture compared to placebo-acupuncture for postoperative nausea and vomiting prophylaxis: A randomised placebo-controlled patient and observer blind trial. Anaesthesia 2004, 59(2):142-149.

Scheider 2006

*Schneider A, Enck P, Streitberger K, Weiland C, Bagheri S, Witte S, et al: Acupuncture treatment in irritable bowel syndrome. Gut 2006, 55(5):649-654.

Schneider A, Enck P, Streitberger K, Joos S, Weiland C, Bagheri S, et al: Spezifische physiologische und unspezifische psychische Effekte bei der Akupunkturtherapie des Reizdarmsyndroms: Ergebnisse einer randomisierten kontrollierten Studie. Deutsche Zeitschrift für Akupunktur 2008, 51(1):8-16.

Schneider A, Weiland C, Enck P, Joos S, Streitberger K, Maser-Gluth C, et al: Neuroendocrinological effects of acupuncture treatment in patients with irritable bowel syndrome. Complementary Therapies in Medicine 2007, 15(4):255-263.
