# Supplementary material for: Quantity, topics, methods and findings of randomised controlled trials published by German university departments of general practice – systematic review
Source: Trials. 2016 Apr 23;17:211. doi: 10.1186/s13063-016-1328-y (PMC4842270; doi:10.1186/s13063-016-1328-y)
Supplement: Additional file 2: — Details of excluded studies. (DOCX 17 kb) [file 13063_2016_1328_MOESM2_ESM.docx]

**Details of excluded studies**

Appendix table 1

Participants/condition, interventions, controls, main outcomes, unit of randomization and sample size (number of clusters/individuals randomized) of planned or ongoing trials (published protocols; n = 17) meeting inclusion criteria

| First author year | Participants, condition or problem addressed | Intervention(s) | Control(s) | Main outcome(s) | Unit random. | Sample size$ |
| --- | --- | --- | --- | --- | --- | --- |
| Altiner 2012a | Respiratory tract infection | 1. Communication training 2. 1 + point-of-care testing | Usual care | Antibiotic prescription rate | Cluster | 188/13160 |
| Altiner 2012b | Patients with at least 3 chronic conditions | Mulitfaceted educational interventions | Usual care | Medication intake, quality of life | Cluster | 48/600§ |
| Barzel 2013 | Chronic stroke (with impaired arm function) | Modified constraint-induced movement therapy | Usual care | Quality of arm and hand use, Wolf motor function test | Cluster | 48/144 |
| Bozorgmehr 2014 | Diabetes type II and ≥ 2 chronic conditions | Network-based care management | Usual care | Diabetes-related self care behaviors | Patient | 582 |
| Drewelow 2012 | Diabetes type II | Educative intervention on patient-centred communication | Usual care | HbA1c, patient participation, quality of life | Cluster | 60/780§ |
| Freund 2011 | COPD, d iabetes type II, chronic heart failure | Practice-based care management | Usual care | Hospitalisation, quality of life, quality of care | Cluster | 130/2210$ |
| Gagyor 2012 | Uncomplicated urinary tract infection | Conditional antibiotic therapy (initially ibuprofen) | Immediate antibiotic therapy | Antibiotic prescription over time and disease burden | Patient | 494 |
| Gensichen 2014 | Panic disorder with or without agoraphobia | CBT oriented psychoeducation and exposure exercises | Usual care | Beck Anxiety Inventory | Cluster | 74/444 |
| Gummersbach 2013 | Women aged 48 to 49 years | New information brochure on mammography screening | Old brochure | Intention to undergo mammography screening | Patient | 346 |
| Jäger 2013 | Mulitmorbid patients with > 4 drugs | Tailored training on medication management in quality circles | Usual care (no special training) | Implementation of three recommendations | Cluster | 4/6800 |
| Löffler 2014a | Antibiotic prescribing in dental practice | Educational seminar for antibiotic prescribing modification | No intervention/usual care | Overall antibiotic prescribing rate | Cluster | 58/46000 |
| Löffler 2014b | Chronically ill elderly hospital patients | Intervention aiming at reducing polypharmacy | Usual care | Health-related quality of life, number of long-term drugs | Cluster | 42/1626 |
| Mortsiefer 2008 | Hypertension | Manual on cardiovascular risk management + outreach visit | Manual only | Calculated cardiovascular risk, single risk factors | Cluster | 92/3146 |
| Schmidt 2010 | Low back pain | Educational intervention according to risk of developing chronic pain | Usual care | Functional capacity, sick leave | Cluster | 40/600 |
| Schmidt 2014 | Sepsis survivors | Discharge management, GP training, telephone management | Usual care | Health-related quality of life, sepsis sequelae | Patient | 290 |
| Siebenhofer 2012 | Indication for oral anticoagulation | Complex intervention (training, case management etc.) | Usual care | Thromboembolia req. hospi-talisation or major bleeding | Cluster | 46/690 |
| Zimmermann 2014 | Anxiety, depression or somatoform disorder | Case management and counseling by advanced practice nurses | Usual care | General Self-Efficacy Scale | Cluster | 20/340 |

$ planned sample sizes; CCBT = cognitive behaviour therapy

Appendix table 2

Participants/condition, interventions, controls, main outcomes, unit of randomization and sample size (number of clusters/individuals randomized) of studies excluded for other reasons (n = 10)

| First author year | | Participants/ condition | | Intervention(s) | | Control(s) | | Main outcome(s) | | Unit random. | Sample size$ |
| --- | --- | --- | --- | --- | --- | --- | --- | --- | --- | --- | --- |
| **Clinical trials with a first author with affiliation to a GP institute at time of publication, but trial planned and performed at other department** | | | | | | | | | | | |
| Joos 2005 | M. Crohn | | Acupuncture | | Minimal acupuncture | | Crohn’s Disease Activity Index | | Patient | | 51 |
| Joos 2006 | Colitis ulcerosa | | Acupuncture + moxibustion | | Sham acupuncture | | Colitis Activity Index | | Patient | | 29 |
| Schneider 2006 | Irritable bowel syndrome | | Acupuncture | | Sham acupuncture | | Quality of life | | Patient | | 43 |
| Schneider 2005 | Postoperative nausea and vomiting | | Acupuncture | | Sham acupuncture | | Nausea, vomiting, perception of bodily sensation | | Patient | | 220 |
| **Short-term experimental (laboratory-like) trials focussing on measurement of physiological measures** | | | | | | | | | | | |
| Doering 2001 | Healthy volunteers | | Kneipp face shower and cold wet pack | | Thermo-indifferent face shower and wet pack | | Cognitive brain function | | Individual  (c-o) | | 24 |
| Doering 2002 | (Probably healthy) volunteers | | CO_2_ dry application | | CO_2_ wet application | | Cerebral hemodynamics | | Individual | | 22 |
| Gavrylyuk 2010 | Healthy volunteers | | Placebo-induced expectation (4 conditions) | | Open placebo | | Pupil size and accommodation | | Individual | | 50 |
| Meissner 2011 | Healthy volunteers | | 1. homeopathy remedy + suggestion  2. placebo + verbal suggestion | | No intervention | | Blood pressure, electrocardio-gram, electrodermal activity | | Individual | | 45 |
| Ronel 2011 | Patients undergoing coronary angiography | | Placebo + verbal suggestion | | Placebo without verbal suggestion | | Diameter of coronary arteries, hemodynamics, distress | | Individual | | 30 |
| **Trial with a last author with affiliation to a German GP institute but not performed in Germany** | | | | | | | | | | | |
| Ludman 2013 | | Depression+diabetes of coronary heart disease | | Self-management support and collaborative care management | | Usual care | | Depressive symptoms, quality of life, HbA1c, blood pressure | | Cluster | 14/214 |

c-o = cross-over design
